# Cell‐specific network analysis of human folliculogenesis reveals network rewiring in antral stage oocytes

**DOI:** 10.1111/jcmm.16315

**Published:** 2021-02-18

**Authors:** Shengran Wang, Yun Gong, Zun Wang, Jonathan Greenbaum, Hong‐Mei Xiao, Hong‐Wen Deng

**Affiliations:** ^1^ Center for System Biology, Data Sciences and Reproductive Health School of Basic Medical Science Central South University Changsha China; ^2^ Tulane Center of Biomedical Informatics and Genomics Deming Department of Medicine Tulane University School of Medicine Tulane University New Orleans LA USA; ^3^ Institute of Reproductive & Stem Cell Engineering School of Basic Medical Science Central South University Changsha China; ^4^ Center of Reproductive Health School of Basic Medical Science Central South University Changsha China; ^5^ Xiangya Nursing School Central South University Changsha China

**Keywords:** antral stage, cell‐specific network, folliculogenesis, single‐cell RNA sequencing

## Abstract

Although previous studies have explored the gene expression profiles of human oocytes and granulosa cells by single‐cell RNA sequencing (scRNA‐seq), the dynamic regulatory network at a single‐cell resolution during folliculogenesis remains largely unknown. We identified 10 functional modules by WGCNA, four of which were significantly correlated with primary/antral oocyte and antral/pre‐ovulatory granulosa cells. Functional enrichment analysis showed that the brown module, which was correlated with antral oocyte, was enriched in oocyte differentiation, and two core subnetworks identified by MCODE were involved in cell cycle (blue subnetwork) and oogenesis (red subnetwork). The cell‐specific network (CSN) analysis demonstrated a distinct gene network structure associated with the antral follicular stage, which was notably different from other developmental stages. To our knowledge, this is the first study to explore gene functions during folliculogenesis at single‐cell network level. We uncovered two potential gene subnetworks, which may play an important role in oocyte function beginning at the antral stage, and further established their rewiring process at intra‐network/whole transcriptome level. The findings provide crucial insights from a novel network perspective to be further explored in functional mechanistic studies.

## 1 INTRODUCTION

Folliculogenesis refers to the biological process by which an ovarian follicle matures through the primordial, primary, secondary, antral and pre‐ovulatory developmental stages.^1^ The follicle is comprised of granulosa cells (GCs), which surround and support the immature oocyte. Follicle growth is rapidly increased during the antral stage, when it becomes more responsive to female hormones (eg follicle‐stimulating hormone), and the end of the development process is marked by separation of the oocyte from the GCs in the pre‐ovulatory stage. Although the large majority of follicles never complete the full development process, a small number produce mature oocytes, which are released during ovulation.

The specific gene expression pattern in different follicle maturation stages and their dynamic changes are noted to be a key factor for folliculogenesis regulation.^2^, ^3^ Previous studies have used single‐cell RNA sequencing (scRNA‐seq) to explore the transcriptomes of the human oocytes and GCs at five follicular stages in vivo and identified candidate secretory biomarkers of ovarian reserve in primordial and primary follicles at gene expression level.^2^ However, the gene interactions and dynamic network changes throughout the folliculogenesis developmental stages remain largely unexplored.

Recently, a cell‐specific network (CSN) analysis approach has been developed to explore the comprehensive gene relationships for scRNA‐seq data from a network viewpoint.^4^ In contrast to traditional network construction, which examines the relationships between genes at the grouped cell level, the CSN method constructs a separate network for each individual cell, thereby accounting for the cell type heterogeneity. This analysis has been efficiently applied to infer key time‐points of gene functions by revealing the network topology changes during human embryo development.^4^ Here, we constructed cell‐specific networks for ovarian follicle development and revealed a potential network rewiring process in antral stage oocytes. Our findings provide a framework of gene relationships during folliculogenesis at the single‐cell level and also lay the foundations to explore characteristic gene functions from a novel network perspective.

## 2 MATERIALS AND METHODS

### 2.1 Data sources and sample information

The data set used in the present study was obtained from the Gene Expression Omnibus (GEO) database (https://www.ncbi.nlm.nih.gov/gds) with Accession Number GSE107746. This data set included the scRNA‐seq gene expression profiles with cell type information of 151 human follicle cells (80 oocytes and 71 GCs) at five different developmental stages.^2^ Cells with fewer than 2400 genes or 500 000 mapped reads were filtered out. In total, 80 oocytes and 71 GCs at five developmental stages passed the filter standards. To ensure the accuracy of estimated gene expression levels, only genes with FPKM > 1 in at least one cell were analysed. An offset value of 1.0 was added for each gene, and the expression levels were log2‐transformed for further analysis. Cell type information is listed in Table S1.

### 2.2 Construction of co‐expression modules

The top 5,000 highly variable genes were selected using the FindVariableFeatures function in Seurat (v3.1.2), and weighted gene co‐expression network analysis (WGCNA) was used to identify functional modules in the co‐expression network.^5^ WGCNA uses soft thresholding to select an appropriate power for constructing the adjacency matrix from the gene correlation matrix. The power amplifies the differences in gene co‐expression and is selected such that the co‐expression network will have a scale‐free topology (β = 14, Figure S2B). The adjacency matrix is used to construct a topological overlap matrix, which is then used as input for hierarchical clustering to identify gene functional modules. We set the minimum module size to be 30 genes and each module was represented by its eigengene, defined as the first principal component of a given module. The average gene significance (GS) was calculated to identify correlation between module eigengenes and a certain cell type, and module membership (MM) was defined as the Pearson correlation between each gene and the module eigengene.

### 2.3 Protein‐protein network (PPI) construction and subnetwork identification

The important WGCNA module genes with GS > 0.2 and MM > 0.8 were used as input to construct the PPI network to assess their inter‐relationships at protein level. The PPI network was constructed by the Search Tool for the Retrieval of Interacting Genes (STRING) database and Cytoscape software. Molecular complex detection (MCODE) was used to identify the most densely connected subnetworks in the PPI network with the following parameters: degree cut‐off = 2, node score cut‐off = 0.2, K‐core value = 2 and MCODE score > 5. The Pearson correlation coefficient between genes in each subnetwork was calculated by using the R package corrplot.

### 2.4 Functional enrichment analysis

Functional enrichment analysis was conducted on four WGCNA gene modules and two PPI subnetworks. Gene Ontology (GO) and Kyoto Encyclopedia of Genes and Genomes (KEGG) analysis were conducted using the R package ClusterProfiler. Only those terms with adjusted *P* value < 0.05 were considered as significant. The top 20 records ranked by adjusted *P* value were extracted when more records were returned.

### 2.5 Cell communication inference

We used an open‐source R package iTALK (https://github.com/Coolgenome/iTALK) to identify and illustrate intercellular signalling communications. This approach was designed to profile the ligand‐receptor (L‐R)–mediated cross‐talk signals from scRNA‐seq data based on a built‐in database, which includes 2648 L‐R pairs classified into four categories: cytokines/chemokines, immune, checkpoint genes, growth factors and others. We ranked the top 100 L‐R pairs based on their expression levels, and the top 20 highly abundant L‐R gene pairs were selected for further visualization.

### 2.6 Immunohistochemistry

We performed immunohistochemistry staining of the ovarian tissue samples of SD rat (8 week) as previously described.^2^ Briefly, the paraffin‐embedded ovarian tissue sections were deparaffinated and rehydrated in xylene and decreasing graded ethanol. Subsequently, the sections were demasked in citrate buffer (pH 6.0) for 20 minutes at 98℃ and cooled down to room temperature and incubated in 0.3% H_2_O_2_ for 10 minutes. The sections were treated overnight at 4°C with primary antibodies, followed by consecutive incubations with biotinylated secondary antibody and streptavidin‐peroxidase conjugate. Colour development was achieved using DAB.

### 2.7 Cell‐specific network construction and network degree matrix transfer

We used MATLAB software to construct a CSN of gene associations for each individual cell in the scRNA‐seq data.^4^ In the CSN of cell *k*, there are *m* gene nodes and the edge corresponding to the pairwise association between genes x and y is estimated by ${\hat{\rho }}_{\mathrm{xy}}^{\left(k\right)}$ (Equation 1). The CSN method can be illustrated as a scatter diagram based on the expression values of genes x and y in different cells (Figure S1), in which each dot represents an individual cell (x‐axis shows the expression values of gene X, and y‐axis shows the expression values of gene Y for cell k). The number of dots (ie cell number) in the green, yellow and intersection boxes is denoted as $n_{x}^{\left(k\right)}$, $n_{y}^{\left(k\right)}$ and $n_{\mathrm{xy}}^{\left(k\right)}$, respectively, where n represents the total cell number in the scatter diagram (ie n = 151). We used the default parameter settings for CSN, where $n_{x}^{\left(k\right)}=n_{y}^{\left(k\right)}=0.1n$, and the coefficient 0.1 denotes the box size.

(1) $${\hat{\rho }}_{\mathrm{xy}}^{\left(k\right)}=\frac{\sqrt{n‐1}\cdot \left(n\cdot n_{\mathrm{xy}}^{\left(k\right)}‐n_{x}^{\left(k\right)}n_{y}^{\left(k\right)}\right)}{\sqrt{n_{x}^{\left(k\right)}n_{y}^{\left(k\right)}\left(n‐n_{x}^{\left(k\right)}\right)\left(n‐n_{y}^{\left(k\right)}\right)}}$$

After hypothesis testing to determine the significance of the edge, the edge weight between genes edge xy (k) was set to either 0 or 1. The network degree matrix (NDM) was then constructed to represent the network features in a lower dimension. For gene x in the network of cell k (Equation 2):

(2) $$NDM_{\mathrm{xk}}=\sum_{y=1,y\neq x}^{m}edge_{\mathrm{xy}}^{\left(k\right)}$$

## 3 RESULTS

### 3.1 Construction of gene co‐expression network

**FIGURE 1:**
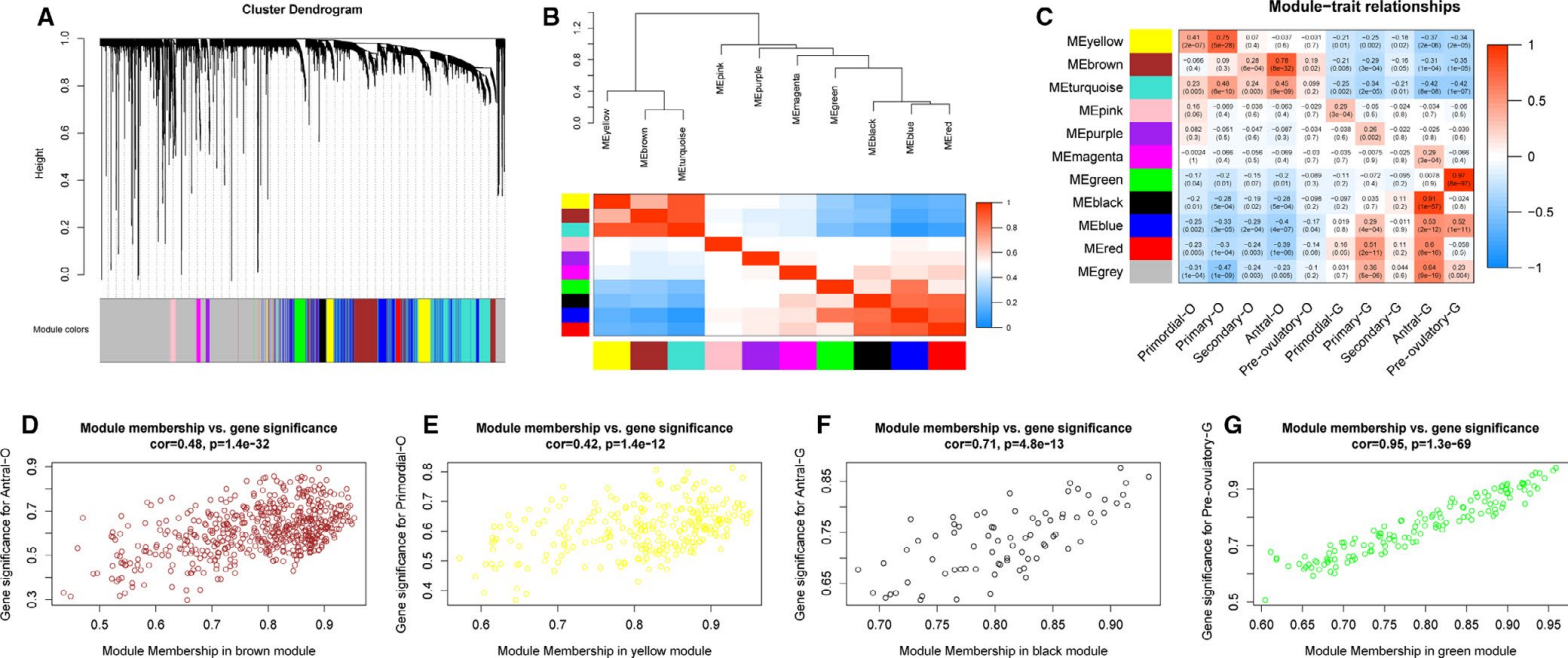
Construction of gene correlation modules. A, The clustering dendrograms of genes. Each gene is represented by one branch, and ten co‐expression modules were constructed and are represented by different colours. B, The eigengene dendrogram and heat map identify groups of correlated eigengenes cross modules. C, Module‐trait relationships. Each row presents a module eigengene, and columns indicate cell type. The corresponding correlation and *P* value have been marked out. D, Scatter plots of module membership vs. gene significance in brown, yellow, black and green modules

Based on hierarchical clustering in WGCNA, the oocytes and GCs were correctly divided into two clusters (Figure S2A). When the power value for constructing the adjacency matrix was set to 12, the independence degree was estimated to be 0.9 and the mean connectivity was close to zero, suggesting a scale‐free topology of the co‐expression network (Figure S2B). WGCNA identified ten distinct functional modules ranging in size from 48 genes (purple module) to 935 genes (turquoise module) (Figure 1A). The eigengene adjacency heat map suggested that these ten modules could be classified into two distinct clusters (Figure 1B).

### 3.2 Quantifying gene co‐expression module‐cell type associations

Modules in the first cluster (including yellow, brown and turquoise modules) were identified to have the highest correlation coefficients between their eigengenes with oocytes; eigengenes of the modules in the other cluster (including pink, purple, magenta, green, black, blue, red modules) had the highest module‐cell type correlation coefficients in GCs (Figure 1B‐C). The yellow and brown modules were more specifically associated with primary and antral stage oocytes, respectively. The black and green modules were more specifically associated with antral and pre‐ovulatory stage GCs, respectively (Figure 1D‐G). These four modules (yellow, brown, black and green) were considered for further downstream analysis.

### 3.3 Functional enrichment analysis

**FIGURE 2:**
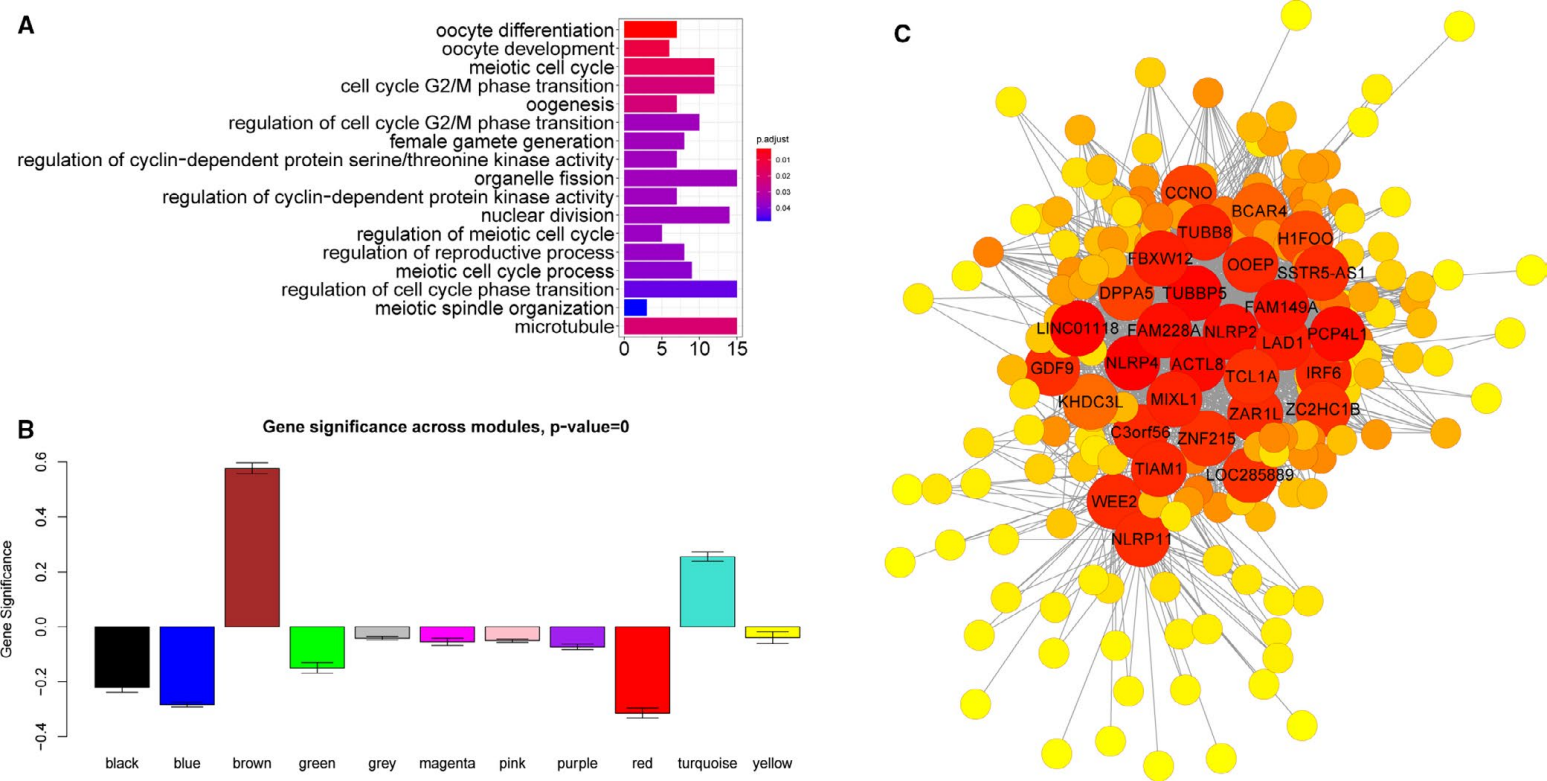
Analysis of the brown module associated with antral stage oocytes. A, GO enrichment results of genes in brown module. B, Gene significance identified as associated with antral stage oocytes crosses modules. C, Co‐expression regulation network in brown module. Red nodes have a high connection degree, and the top 30 degree nodes have been annotated with gene symbols

GO enrichment analysis was performed to uncover the biological function of genes in the four interesting modules (Figure 2A, Figure S3A‐C). The analysis revealed several specifically enriched biological processes related to oocyte development and meiosis (eg GO0009994: oocyte differentiation, GO0048599: oocyte development, GO0051321: meiotic cell cycle, GO0048477: oogenesis, GO0051445: regulation of meiotic cell cycle and GO0000212: meiotic spindle organization) that were only associated with antral stage oocytes (*P* adjusted < 0.05; Figure 2A). In addition, the brown module had the highest gene significance score (reflecting how strongly the module gene expression values correlate with a certain cell type) across modules (Figure 2B), indicating that genes in the brown module were predominantly up‐regulated in this cell type. The top 30 high‐degree genes in the brown (Figure 2C), yellow, black and green modules (Figure S3D‐F) were denoted in their respective networks.

### 3.4 Oocyte and granulosa cell communications

**FIGURE 3:**
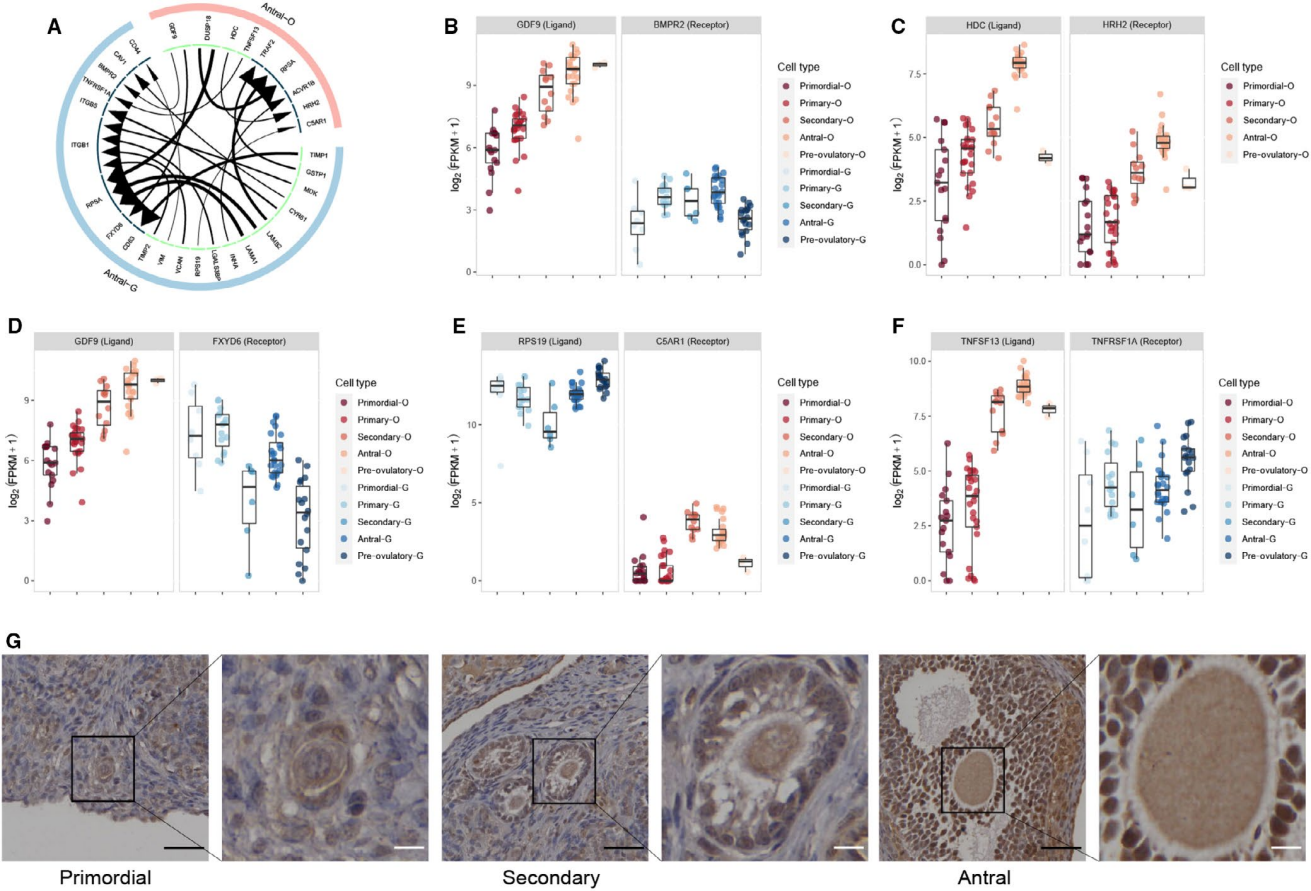
Analysis of cell communication in antral stage follicles. A, Circos plots show the top 20 highly expressed ligand‐receptor interactions in antral stage follicle. B. The expression of *GDF9* (ligand)‐*BMPR2* (receptor) gene pair. C, The expression of *HDC* (ligand)‐*HRH2* (receptor) gene pair. D, The expression of *GDF9* (ligand)‐*FXYD6* (receptor) gene pair. E, The expression of *RPS19* (ligand)‐*C5AR1* (receptor) gene pair. F, The expression of *TNFSF13* (ligand)‐*TNFRSF1A* (receptor) gene pair. G, Immunohistochemistry staining of *HDC* in primordial, secondary and antral stage oocytes. The scale bars represent 40 μm in low‐magnification view and 10 μm in high‐magnification view

We used iTALK to characterize the L‐R–mediated intercellular communication and list the top 100 L‐R pairs in antral stage oocytes and GCs (Table S2). Figure 3A displayed the top 20 highly expressed L‐R interactions. In addition, the box plots showed the expression levels of five L‐R pairs that were included in the brown module (Figure 3B‐F). Most of these genes have high expression levels in the antral stage. Several genes including *BMPR2*, *HDC*, *HRH2* and *TNFSF13* even have the highest expression levels during other developmental stages. The expression levels of *HDC* in different stage oocytes were supported by immunofluorescence labelling (Figure 3G).

### 3.5 Pivotal subnetwork identification of genes associated with antral stage oocyte

**FIGURE 4:**
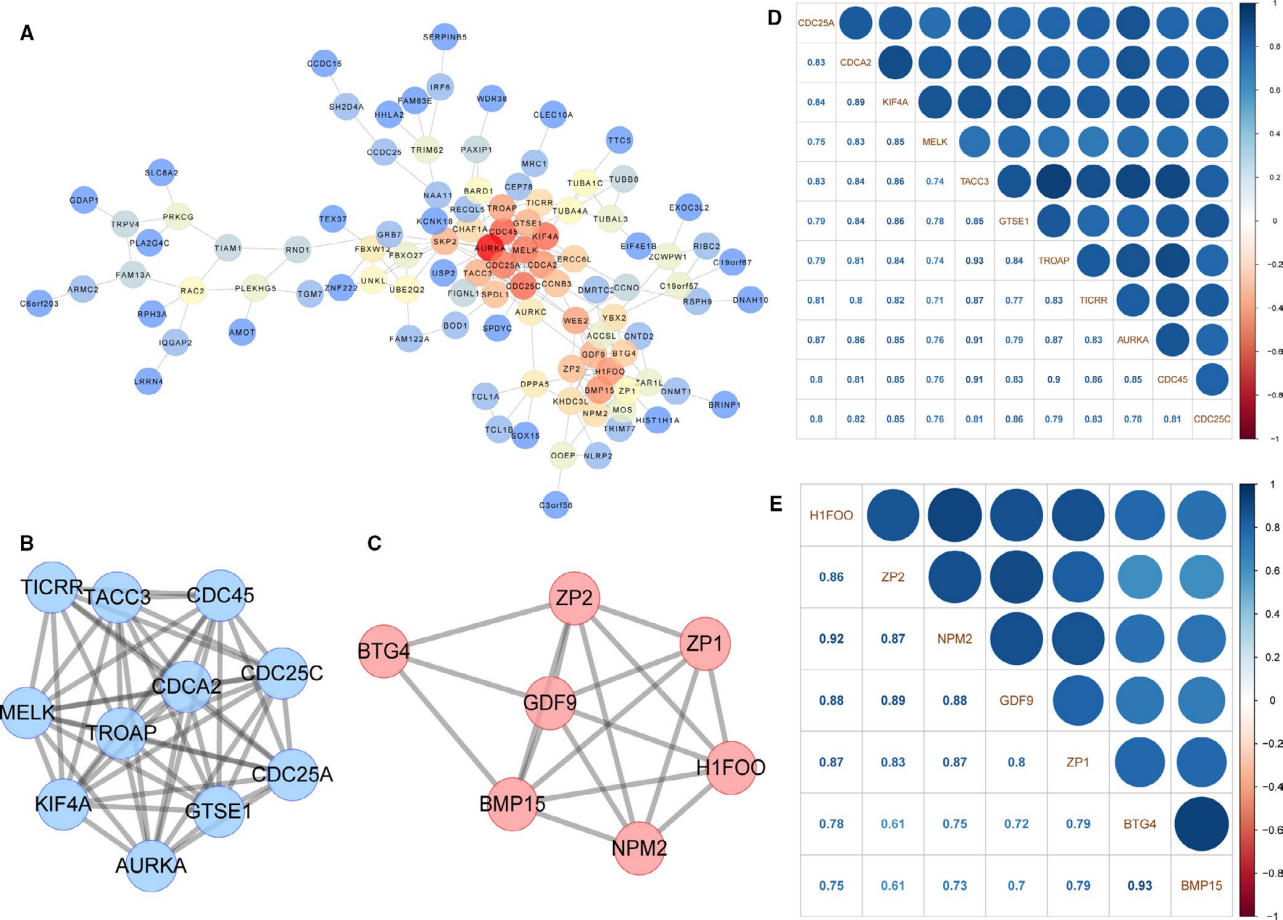
Visualization of PPI network analysis result. A, PPI network of brown module genes with GS > 0.5 and MM > 0.8. B, A subnetwork screened by MCODE, MCODE score = 10.2. C, Another subnetwork screened by MCODE, MCODE score = 6.0. D, Correlation between the blue subnetwork genes. E, Correlation between the red subnetwork genes

**FIGURE 5:**
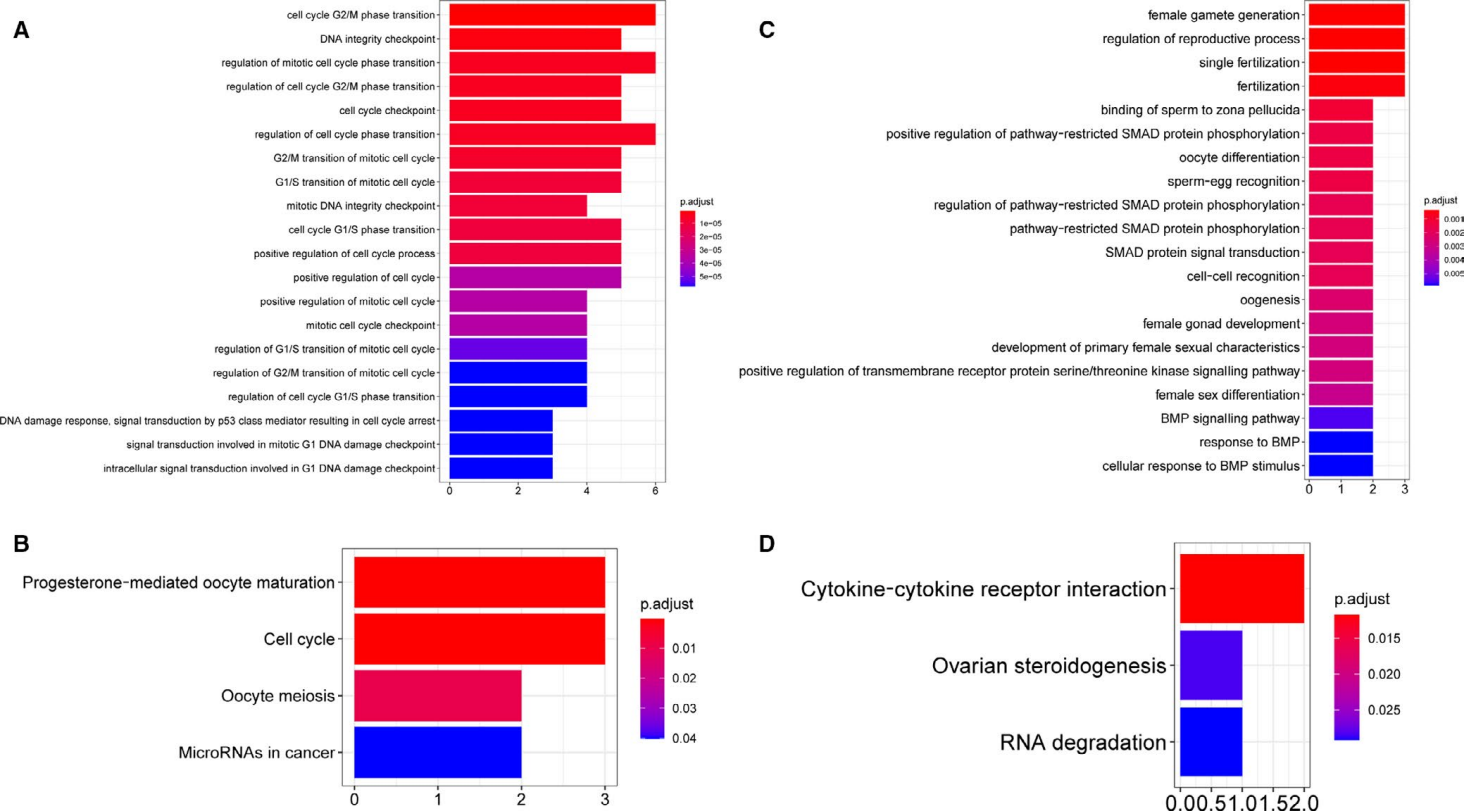
Enrichment analysis of two subnetwork genes. A, Top 20 GO enrichment analysis results of the blue subnetwork genes. B, KEGG enrichment analysis results of the blue subnetwork genes. C, Top 20 GO enrichment analysis results of the red subnetwork genes. D, KEGG enrichment analysis results of the red subnetwork genes

Genes with both high gene significance (GS > 0.2) and high module membership (MM > 0.8) in the brown module were used to construct the PPI network (Figure 4A), and MCODE was used to extract the pivotal subnetworks as hub modules. We identified two hub modules in the original PPI network (Figure 4B‐C). The blue module (MCODE score = 10.6) consisted of eleven genes, including *CDC25C* and *KIF4A*. The red module (MCODE score = 6.0) consisted of seven genes including *BMP15*, *GDF9*, *ZP1* and ZP2. Furthermore, genes in these two subnetworks were significantly correlated with each other (Figure 4D‐E). GO and KEGG pathway analysis revealed that the blue subnetwork genes were mainly enriched in GO0044839 (cell cycle G2/M phase transition), GO0031570 (DNA integrity checkpoint), GO0000075 (cell cycle checkpoint) and hsa04114 (oocyte meiosis; Figure 5A‐B), whereas red subnetwork genes were highly enriched in GO0009994 (oocyte differentiation), GO0048477 (oogenesis), GO0030509 (BMP signalling pathway), GO0048599 (oocyte development) and hsa04913 (ovarian steroidogenesis; Figure 5C‐D).

### 3.6 CSN rewiring in antral stage oocyte

**FIGURE 6:**
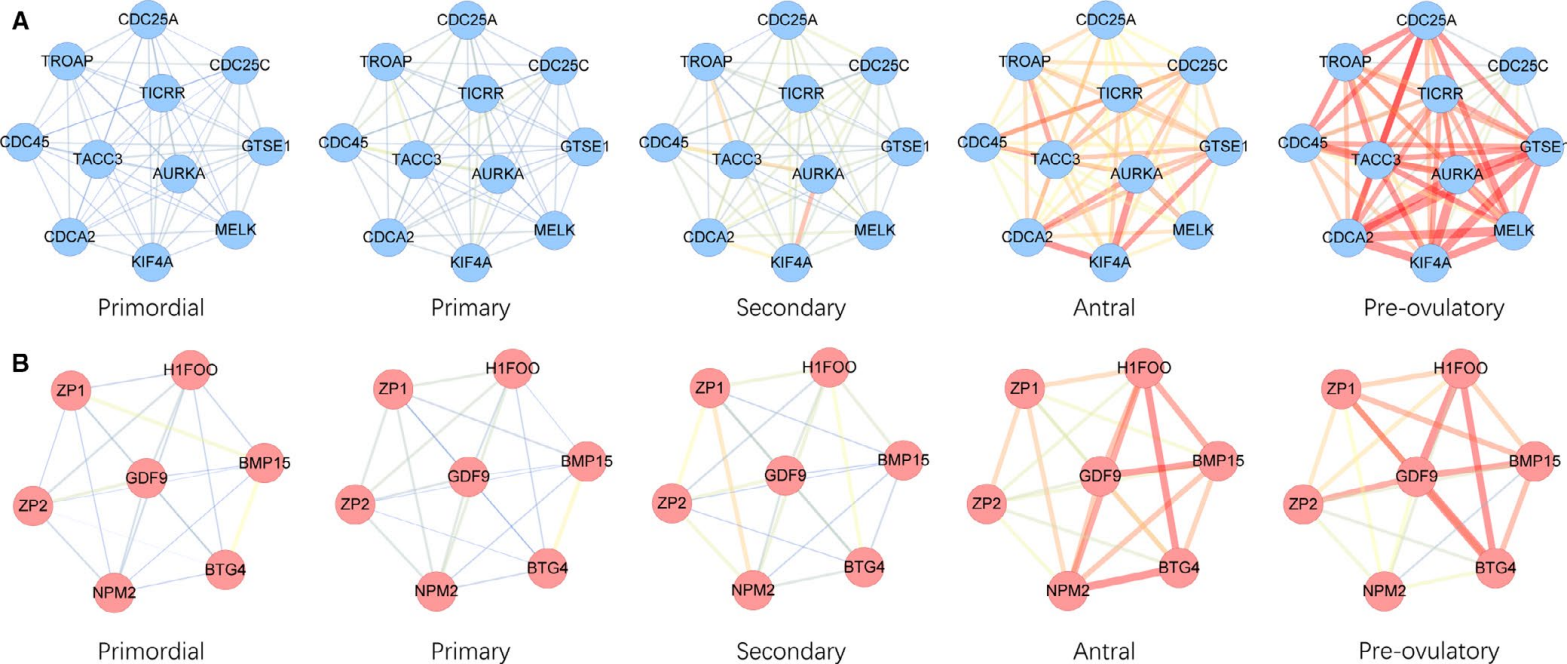
CSN construction among two pivotal subnetworks. A, CSN of the blue subnetwork. Edge colour represents mean connection score in each cell type from blue (low) to red (high). B, CSN of the red subnetwork. Edge colour represents mean connection score in each cell type from blue (low) to red (high)

We performed the network rewiring analysis on the two identified subnetworks. We observed that the subnetwork genes were weakly interconnected in primordial, primary and secondary stages, and that the connections between these genes become much stronger in the antral stage. For instance, in the antral stage, strong connections were established between *GTSE1*, *KIF4A*, *CDCA2*, *NPM2*, *H1FOO*, *BMP15* and other genes. In the antral stage, the gene associations were strongest between *CDC25C*, *NPM2* and *ZP1*, although the connections between all the genes involved in these two subnetworks were greatly strengthened during this stage. These findings based on the CSN algorithm suggest a drastic network rewiring process during folliculogenesis (Figure 6).

### 3.7 Dynamic changes in network degree along folliculogenesis

**FIGURE 7:**
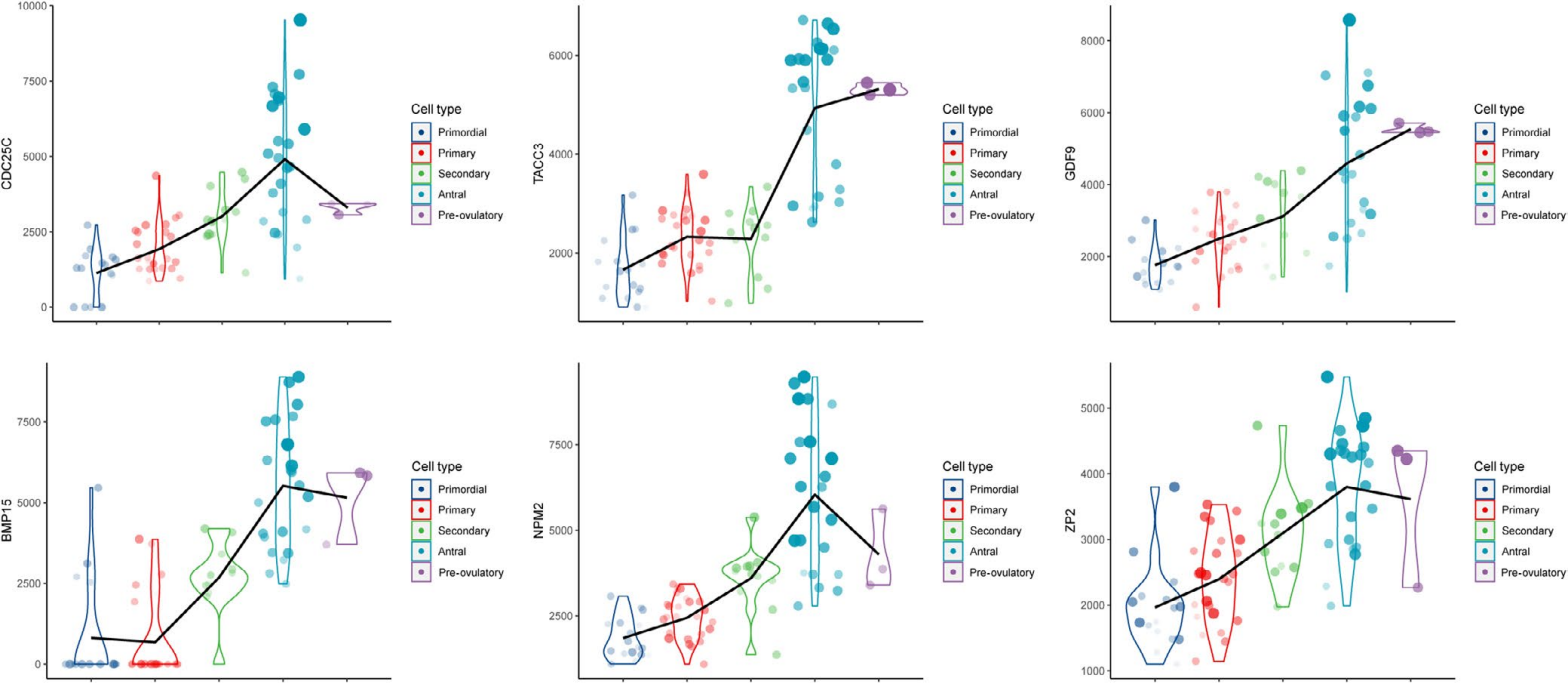
NDM of two subnetwork genes. Differential network degrees of two subnetwork genes in five follicle stages. Point size represents the total connection scores among the subnetworks that the gene belonged to

To explore the gene connections on the whole transcriptome level, we calculated network degrees of each gene in the two subnetworks. The network degrees of *CDC25C*, *TACC3*, *TICRR*, *TROAP*, *NPM2*, *BMP15* and *ZP2* approached their peaks in the antral stage (Figure 7). Additionally, most other genes in the two subnetworks also demonstrated the highest degree in the antral oocytes with the exception of *AURKA*, *KIF4A* and *MELK* (Figure S4). The largest differences in NDM trends across different subnetwork genes were observed in the pre‐ovulatory stage at the conclusion of folliculogenesis.

## 4 DISCUSSION

With the development of single‐cell sequencing technology, new algorithms have provided an unprecedented opportunity to identify gene associations/networks at the single‐cell resolution level.^4^ By integrating the classical WGCNA into the novel CSN analysis, our research aimed to explore the gene functions of follicles, especially at antral stage. Antral stage is an important period in folliculogenesis as antral follicle status plays a critical role in ovarian functions and influence reproductive abilities.^6^, ^7^, ^8^ The biomarkers specific to the antral stage follicle are also representative indicators for the prediction of ovarian response in fertility treatments.^8^, ^9^

In this study, ten co‐expression gene modules were constructed using the most highly variable genes in 151 oocytes and GCs.^2^ Four modules were detected as significantly correlated with a specific oocyte or GC subtype. GO enrichment analysis across the four gene modules identified one module (brown module), which was mainly enriched in meiotic cell cycle and oocyte differentiation, underlying their potential roles in oogenesis. We then confirmed that this functional module had the highest gene significance value in antral stage oocytes, and further used Cytoscape software to show the memberships in this module. The module genes were then sorted based on their connection degree.

The genes with the highest degree connectivity were *NLRP4*, *NLRP2* and *NLRP11*. The NLRP (nucleotide‐binding oligomerization domain, leucine‐rich repeat and pyrin domain‐containing) gene family has previously been reported to be involved in reproductive functions.^10^, ^11^, ^12^ Various NLRPs were shown to have stage‐dependent expression level during human pre‐implantation development,^13^ and they are duplicated and functionally diversified in mammalian reproductive systems.^14^, ^15^, ^16^ Tubulin beta‐eight class VIII (*TUBB8*) is a subtype of β‐tubulin that only exists in primates, and mutations in *TUBB8* are responsible for human oocyte meiotic arrest.^17^ As most of these high‐degree genes have previously been reported to be involved in reproductive function,^18^, ^19^, ^20^, ^21^, ^22^, ^23^ we selected the brown module genes for further downstream analysis.

Reciprocal communications between human oocytes and GCs played key roles in folliculogenesis.^24^, ^25^, ^26^ We predicted interactions of GCs‐oocytes, oocytes‐GCs, oocytes‐oocytes and GCs‐GCs in antral stage follicles and showed the top five L‐R pairs involving brown module genes. Growth differentiation factor 9 (*GDF9*) has been shown to act through miR‐375 to affect *BMPR2* expression and Smad signalling pathway activation,^27^ which ultimately affected the proliferation, spread and apoptosis of bovine cumulus cells.^28^ Exploring the effect of the *GDF9*‐miR‐375‐*BMPR2* molecular pathway on human antral stage follicles may be a valuable future research direction. Another interesting identified gene pair was *HDC* (ligand) and *HRH2* (receptor), which were specifically highly expressed in antral follicles. Transplanted imaginal discs homozygous for an hdc mutation were found to affect oogenesis in the recipient females,^29^ although the specific function of *HDC* and *HRH2* remains largely undefined. Our findings provide novel insight into this gene pair interaction, which may be related to autocrine signalling in oocytes.

Hao et al^4^ presented a novel algorithm to construct a CSN for individual cells from scRNA‐seq transcriptomic profiles. This method transforms the 'unstable' gene expression form to a more 'stable' gene association form. This is the first technique to enable the identification of gene associations/network at a single‐cell resolution level. We applied the CSN approach to the noteworthy PPI subnetworks and demonstrated that many connections, which were weak in the early development stages, became significantly stronger in the antral stage. Furthermore, network degree connectivity of many subnetwork genes approached their peaks in the oocytes at this period. These results indicate a drastic network rewiring process during the antral stage and further strengthen the evidence that the antral stage may be the key time‐point for the function of the genes in these subnetworks.

Oocyte nucleolus is the most prominent subcellular organelle in the oocyte, and the maternal nucleolus is essential for early embryonic development in mammals.^30^ Proteomic analysis identified nucleoplasmin 2 (*NPM2*) as a dominant component of the oocyte nucleolus.^31^ Additionally, oocytes in knockout mice lacking Npm2 (Npm 2^‐/‐^) could not be transformed into surrounded nucleolus (SN) configuration.^32^ Furthermore, the expression of *NPM2* alone sufficed to reconstitute the nucleolar structure in enucleated embryos, and rescued their full‐term development.^31^ Our result indicates that antral stage oocytes may have special nucleus actions in nucleolus via *NPM2* and thus establish a foundation for further cytobiology studies.

*TACC3* is a core component of the liquid‐like meiotic spindle domain (LISD) assembly. Mammalian oocytes segregated chromosomes with a specialized non‐centrosomal microtubule spindle through LISD, which was quite different from both somatic cells and male germ cells. *TACC3*(ΔNT)‐*TACC3*–depleted oocytes exhibited serious spindle bipolarization delay with spindle instability and fragmentation.^33^ The drastic changes in *TACC3* in the CSN level may imply that the oocytes may assist in the preparation for the subsequent meiotic division process. Further research should provide insight into the detailed mechanisms of these drastic changes.

The replication helicase is comprised of CMG (CDC45, MCM2‐7 and GINS) in eukaryotic cells.^34^ Treslin (encoded by *TICRR*), which orchestrates assembly of the CMG in human cells, is essential for incorporation of CDC45 into the replicative helicase and helps to trigger the initiation of DNA replication.^35^ GTSE1 is an upstream regulator of microtubule stability, chromosome alignment and spindle pole integrity mediated by KIF4A.^36^, ^37^ The cells depleted of GTSE1 display hyper‐stabilized spindle microtubules, which in turn diminish accumulation of the chromokines in KIF4A,^38^ and depletion of KIF4A induces multipolar spindles.^37^ GDF9 and BMP15 play vital roles in follicular growth, atresia, ovulation, fertilization, reproduction and maintenance. Numerous studies in both humans and mice have demonstrated a synergy between BMP15 and GDF9,^39^, ^40^ which is primarily mediated by the bone morphogenetic protein type II receptor (BMPR2).^41^ Human ZP is composed of four glycosylated proteins, ZP1‐ZP4, which are synthesized, processed, secreted and assembled into long, cross‐linked fibrils by growing oocytes. A ZP2‐ZP3 dimer is located periodically along ZP fibrils that are cross‐linked by ZP1, a protein with a proline‐rich N terminus.^42^ Mutant ZP1 proteins that fail to interact with either ZP2 or ZP3 will cause oocyte degeneration.^43^ Our results may provide clues for such reported interactions and other undefined interactions distinctive to antral stage oocytes. We note that the general network degrees of these genes were not strictly related to the total connection scores among the subnetworks they belong to. The dynamic tendency of these genes varies, especially in the pre‐ovulatory stage. Although these results may stem from the specific connection state, another factor that must be considered is the insufficient pre‐ovulatory oocyte numbers (only three cells). More cells are needed to get the unbiased expression information, which directly influenced the CSN connections and network degree values. Furthermore, validation of certain molecular functions should be performed to unveil the complex roles of the genes in the CSN during follicular development.

In summary, our analysis identified two pivotal gene subnetworks, which may play an important role in antral stage oocytes. Genes in these subnetworks demonstrated a drastic intra‐network/whole transcriptome rewiring process that began in the antral stage. This is the first study to identify the gene associations/network for oogenesis at single‐cell resolution, and it establishes the foundations to reveal advanced mechanisms in antral stage folliculogenesis in a new perspective based on the cell‐specific network.

## CONFLICT OF INTEREST

The authors have declared no conflict of interest.

## AUTHOR CONTRIBUTIONS

**shengran wang:** Formal analysis (lead); Validation (lead); Visualization (lead); Writing‐original draft (lead); Writing‐review & editing (supporting). **yun gong:** Conceptualization (equal); Writing‐review & editing (equal). **zun wang:** Conceptualization (equal); Writing‐review & editing (equal). **Jonathan Greenbaum:** Conceptualization (equal); Writing‐review & editing (equal). **hongmei xiao:** Funding acquisition (equal); Writing‐review & editing (lead). **hongwen deng:** Funding acquisition (equal); Writing‐review & editing (lead).

## Supporting information

Fig S1

Fig S2

Fig S3

Fig S4

Table S1

Table S2

## Data Availability

The scRNA‐seq data used in this study were available from the GEO data sets with Accession Number GSE107746.
