# Supplementary figures and images for: Cell‐specific network analysis of human folliculogenesis reveals network rewiring in antral stage oocytes
Source: J Cell Mol Med. 2021 Feb 18;25(6):2851–60. doi: 10.1111/jcmm.16315 (PMC7957178; doi:10.1111/jcmm.16315)

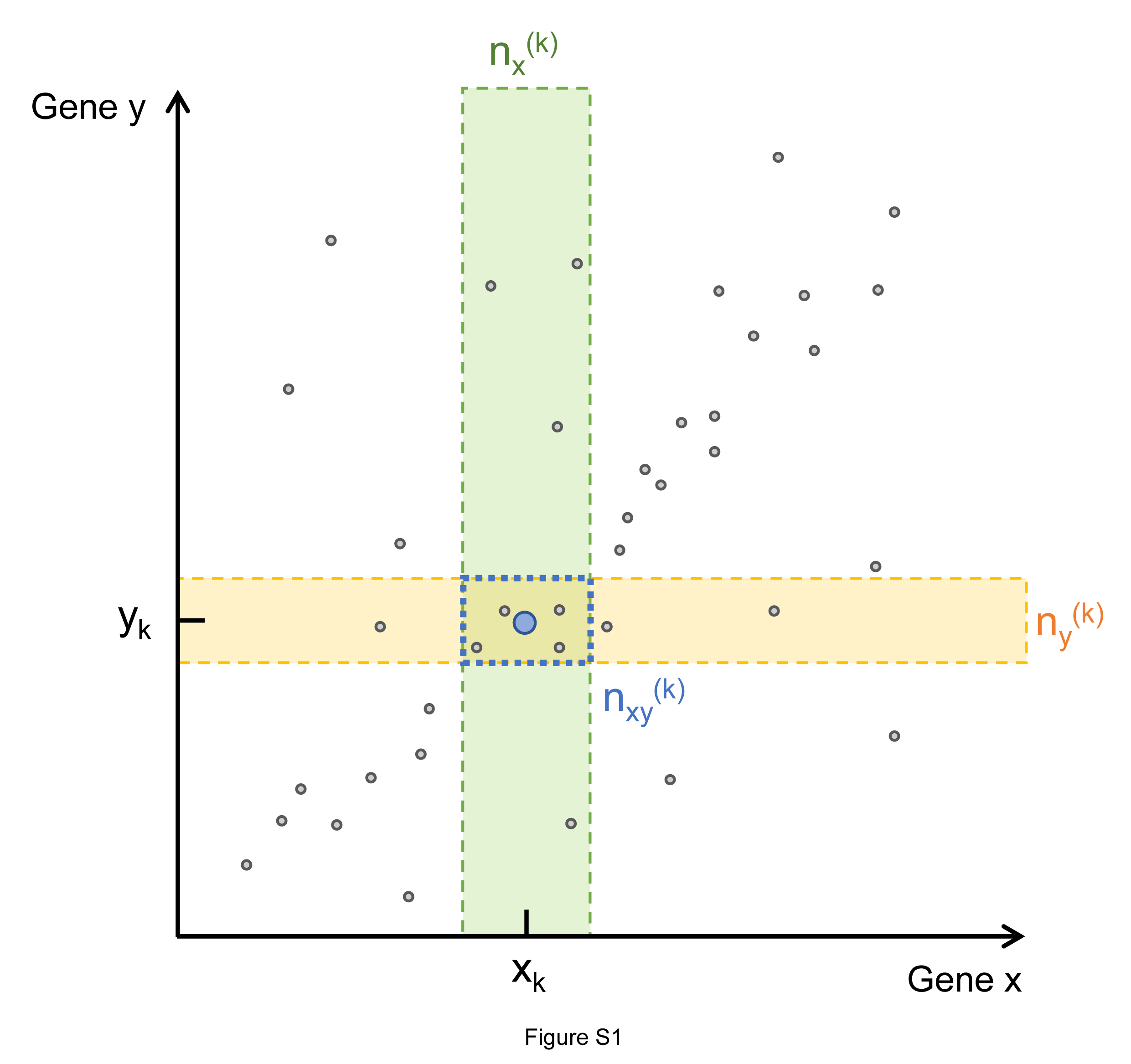

Supplement: Supplementary file 1 — Fig S1 [file JCMM-25-2851-s001.tif]

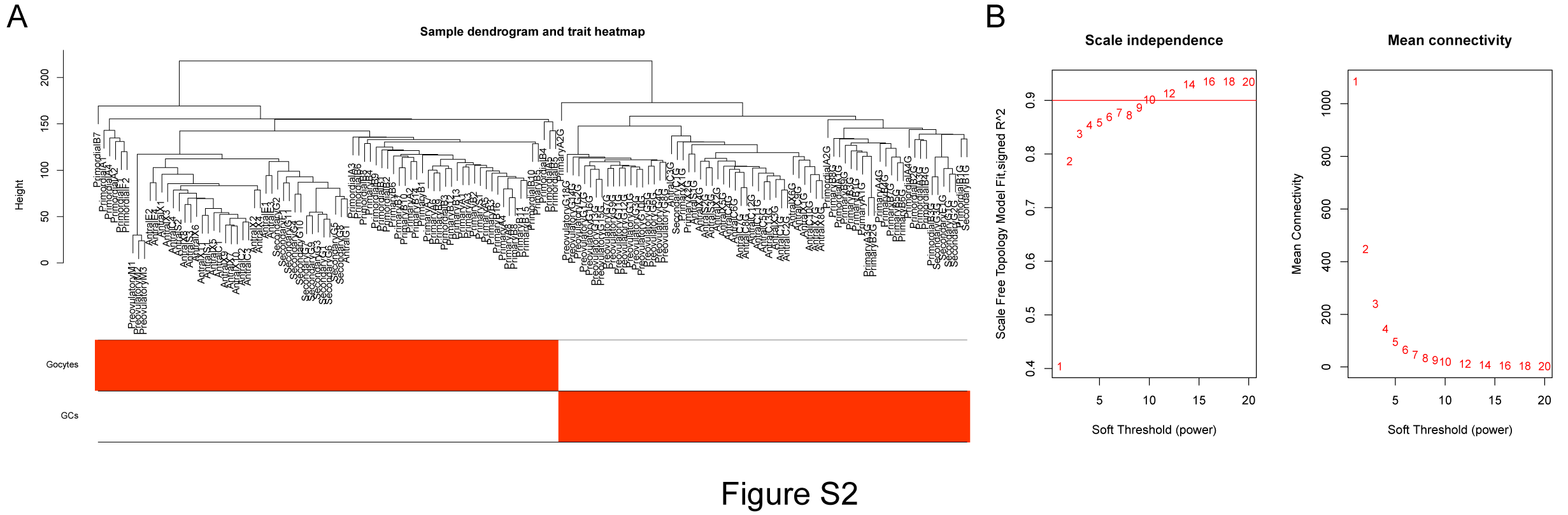

Supplement: Supplementary file 2 — Fig S2 [file JCMM-25-2851-s004.tif]

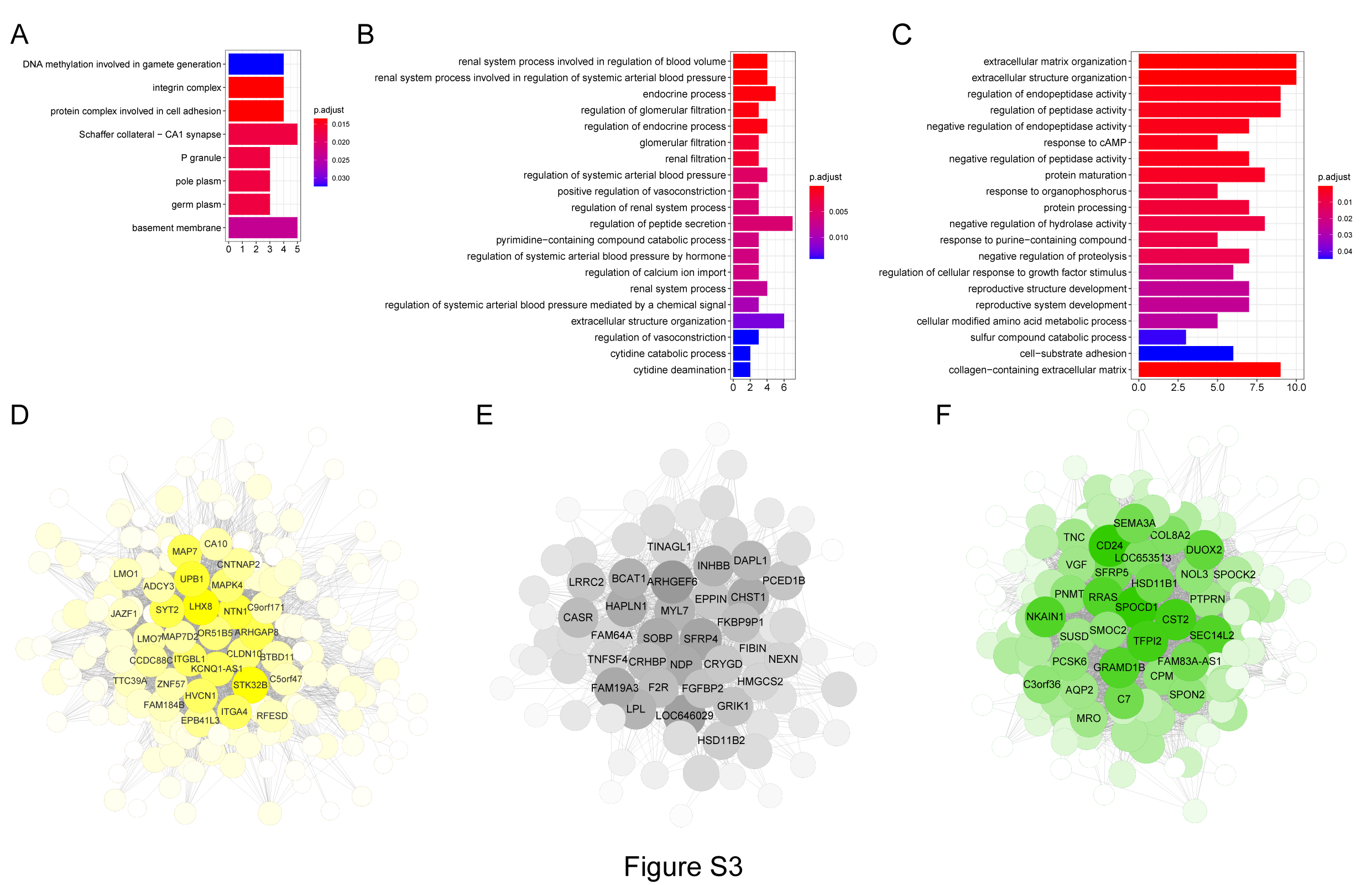

Supplement: Supplementary file 3 — Fig S3 [file JCMM-25-2851-s005.tif]

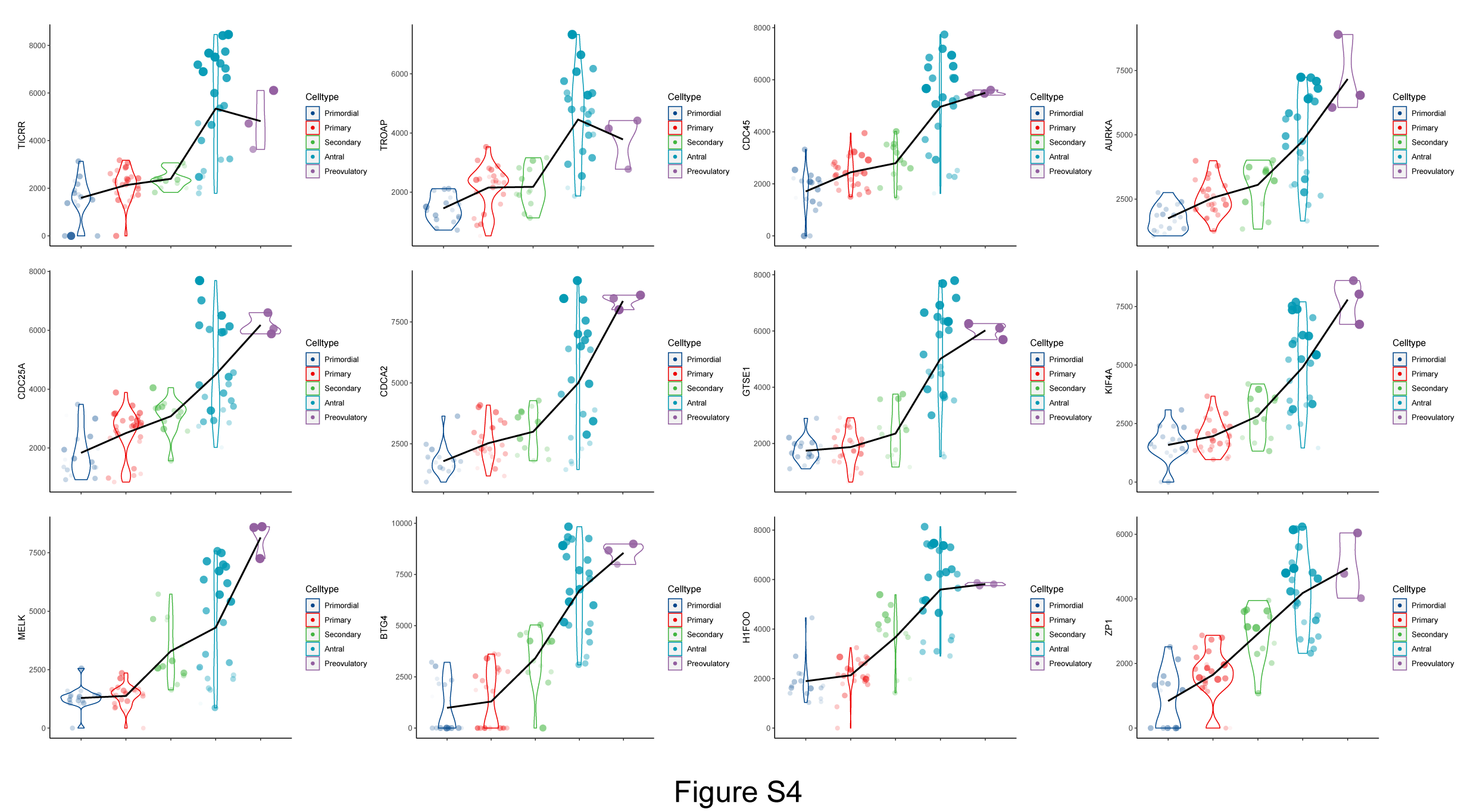

Supplement: Supplementary file 4 — Fig S4 [file JCMM-25-2851-s002.tif]
