# Supplementary material for: Cell‐specific network analysis of human folliculogenesis reveals network rewiring in antral stage oocytes
Source: J Cell Mol Med. 2021 Feb 18;25(6):2851–60. doi: 10.1111/jcmm.16315 (PMC7957178; doi:10.1111/jcmm.16315)
Supplement: Supplementary file 5 — Table S1 [file JCMM-25-2851-s003.pdf]

Table S1 Cell type information of GSE107746 dataset.

Cell type	Cell number
Oocytes (Primordial)	17
Oocytes (Primary)	25
Oocytes (Secondary)	12
Oocytes (Antral)	23
Oocytes (Preovulatory)	3
GCs (Primordial)	8
GCs (Primary)	15
GCs (Secondary)	6
GCs (Antral)	24
GCs (Preovulatory)	18
