# Supplementary material for: Cell‐specific network analysis of human folliculogenesis reveals network rewiring in antral stage oocytes
Source: J Cell Mol Med. 2021 Feb 18;25(6):2851–60. doi: 10.1111/jcmm.16315 (PMC7957178; doi:10.1111/jcmm.16315)
Supplement: Supplementary file 6 — Table S2 [file JCMM-25-2851-s006.pdf]

Table S2 Top 100 highly expressed ligand-receptor interactions in antral stage follicle.

Rank	Ligand	Receptor	Cell from	Cell to	Communication type
1	TIMP1	CD63	GCs	GCs	other
2	GSTP1	TRAF2	GCs	Oocytes	other
3	GDF9	FXYD6	Oocytes	GCs	other
4	DUSP18	RPSA	Oocytes	GCs	other
5	MDK	ITGB1	GCs	GCs	other
6	CYR61	ITGB5	GCs	GCs	other
7	DUSP18	RPSA	Oocytes	Oocytes	other
8	LAMB2	RPSA	GCs	GCs	other
9	LAMA1	RPSA	GCs	GCs	other
10	INHA	ACVR1B	GCs	Oocytes	other
11	LGALS3BP	ITGB1	GCs	GCs	other
12	HDC	HRH2	Oocytes	Oocytes	other
13	TNFSF13	TNFRSF1A	Oocytes	GCs	other
14	LAMB2	RPSA	GCs	Oocytes	other
15	RPS19	C5AR1	GCs	Oocytes	other
16	GDF9	BMPR2	Oocytes	GCs	other
17	CYR61	CAV1	GCs	GCs	other
18	VCAN	ITGB1	GCs	GCs	other
19	VIM	CD44	GCs	GCs	other
20	TIMP2	ITGB1	GCs	GCs	other
21	LAMA1	RPSA	GCs	Oocytes	other
22	GNAS	ADCY9	GCs	Oocytes	other
23	CALM1	PTPRA	Oocytes	Oocytes	other
24	PTDSS1	SCARB1	Oocytes	GCs	other
25	TNFSF13	SDC2	Oocytes	GCs	other
26	B2M	TFRC	GCs	GCs	other
27	FST	BMPR2	GCs	GCs	other
28	GNAI2	CAV1	GCs	GCs	other
29	GNAI2	UNC5B	GCs	GCs	other
30	PSAP	GPR37	GCs	Oocytes	other
31	RTN4	RTN4R	GCs	Oocytes	other
32	INHBA	ENG	GCs	Oocytes	other
33	FN1	ITGB1	GCs	GCs	other
34	PTDSS1	JMJD6	Oocytes	GCs	other
35	ARF1	INSR	GCs	GCs	other
36	INHBB	ACVR1B	GCs	Oocytes	other
37	GAS6	AXL	GCs	GCs	other
38	INHBA	BAMBI	GCs	GCs	other
39	PTDSS1	JMJD6	Oocytes	Oocytes	other
40	GSTP1	TRAF2	GCs	GCs	other
41	DUSP18	ITGB1	Oocytes	GCs	other

42	CALM2	INSR	Oocytes	GCs	other
43	THBS1	CD36	GCs	Oocytes	other
44	LAMB1	ITGB1	Oocytes	GCs	other
45	FN1	ITGB1	Oocytes	GCs	other
46	RARRES2	GPR1	Oocytes	GCs	other
47	COL6A1	ITGB1	GCs	GCs	other
48	THBS1	SCARB1	GCs	GCs	other
49	GNAI2	F2R	GCs	GCs	other
50	THBS1	ITGB1	GCs	GCs	other
51	PTDSS1	SCARB1	GCs	GCs	other
52	BDNF	NGFRAP1	Oocytes	GCs	other
53	VEGFA	ITGB1	GCs	GCs	growth factor
54	CALM2	INSR	GCs	GCs	other
55	ARF1	INSR	Oocytes	GCs	other
56	MDK	SDC4	GCs	GCs	other
57	RPS19	C5AR1	Oocytes	Oocytes	other
58	ITGB3BP	ITGB5	Oocytes	GCs	other
59	CYR61	ITGB5	GCs	Oocytes	other
60	PRSS23	TMEM222	GCs	GCs	other
61	CALM1	PTPRA	GCs	Oocytes	other
62	RTN4	RTN4R	Oocytes	Oocytes	other
63	GNAI2	IGF1R	GCs	GCs	other
64	GNAS	ADCY7	GCs	Oocytes	other
65	CALM1	INSR	Oocytes	GCs	other
66	CALM1	PTPRA	Oocytes	GCs	other
67	INHBA	ACVR1B	GCs	Oocytes	other
68	CALM2	PPAPDC2	Oocytes	Oocytes	other
69	SAA1	SCARB1	Oocytes	GCs	other
70	ADAM17	ITGB1	GCs	GCs	other
71	VEGFA	GPC1	GCs	GCs	growth factor
72	GDF9	TGFBR1	Oocytes	Oocytes	other
73	GNAI2	ADCY9	GCs	Oocytes	other
74	PTDSS1	JMJD6	GCs	GCs	other
75	CALM2	PPAPDC2	GCs	Oocytes	other
76	CALM1	HMMR	Oocytes	Oocytes	other
77	CALM2	KCNQ1	Oocytes	GCs	other
78	HSP90B1	ASGR1	GCs	Oocytes	other
79	GNAI2	S1PR1	GCs	GCs	other
80	PTDSS1	JMJD6	GCs	Oocytes	other
81	GNAI2	CAV1	Oocytes	GCs	other
82	GNAI2	UNC5B	Oocytes	GCs	other
83	PTN	PLXNB2	Oocytes	GCs	other
84	LAMC1	ITGB1	GCs	GCs	other
85	CALM1	PPAPDC2	Oocytes	Oocytes	other

86	PRSS23	TMEM222	GCs	Oocytes	other
87	BDNF	NGFRAP1	GCs	GCs	other
88	COL4A1	ITGB1	GCs	GCs	other
89	MDK	LRP1	GCs	GCs	other
90	NDP	LGR4	GCs	GCs	other
91	SPINT1	ST14	Oocytes	Oocytes	other
92	BMP6	BMPR2	Oocytes	GCs	other
93	CALM2	KCNQ1	GCs	GCs	other
94	CYR61	ITGAV	GCs	GCs	other
95	SEMA7A	ITGB1	GCs	GCs	other
96	GDF9	BMPR1A	Oocytes	Oocytes	other
97	ICAM1	CAV1	Oocytes	GCs	other
98	LAMC3	ITGB1	GCs	GCs	other
99	CALR	ITGAV	GCs	GCs	other
100	GNAI2	IGF1R	GCs	Oocytes	other
